# The effect of bovine BST2A1 on the release and cell-to-cell transmission of retroviruses

**DOI:** 10.1186/s12985-017-0835-0

**Published:** 2017-09-06

**Authors:** Zhibin Liang, Yang Zhang, Jie Song, Hui Zhang, Suzhen Zhang, Yue Li, Juan Tan, Wentao Qiao

**Affiliations:** 1 Key Laboratory of Molecular Microbiology and Technology, Ministry of Education, College of Life Sciences, Nankai University, Tianjin, 300071 China; 2 College of Life Sciences, Nankai University, 94 Weijin Rd, Tianjin, 300071 China

**Keywords:** bBST2A1, HIV-1, PFV, BFV, BIV, Virus release, Cell-to-cell transmission

## Abstract

**Background:**

Human BST2 (hBST2, also called Tetherin) is a host restriction factor that blocks the release of various enveloped viruses. BST2s from different mammals also possess antiviral activity. Bovine BST2s (bBST2s), bBST2A1 and bBST2A2, reduce production of cell-free bovine leukemia virus (BLV) and vesicular stomatitis virus (VSV). However, the effect of bBST2 on other retroviruses remains unstudied.

**Results:**

Here, we studied the antiviral activity of wildtype and mutant bBST2A1 proteins on retroviruses including human immunodeficiency virus type 1 (HIV-1), prototypic foamy virus (PFV), bovine foamy virus (BFV) and bovine immunodeficiency virus (BIV). The results showed that wildtype bBST2A1 suppressed the release of HIV-1, PFV and BFV. We also generated bBST2A1 mutants, and found that GPI anchor and dimerization, but not glycosylation, are essential for antiviral activity of bBST2A1. Moreover, unlike hBST2, bBST2A1 displayed no inhibitory effect on cell-to-cell transmission of PFV, BFV and BIV.

**Conclusions:**

Our data suggested that bBST2A1 inhibited retrovirus release, however, had no effect on cell-to-cell transmission of retroviruses.

## Background

Bone marrow stromal cell antigen 2 (BST2, also called tetherin) blocks the release of *vpu*-deficient human immunodeficiency virus type 1 (HIV-1) [26, 32] by directly tethering the viral particles to the cell surface [28]. This anti-viral function of BST2 is attributed to its topology, which consists of an N-terminal cytoplasmic tail (CT), a single transmembrane domain (TM), an extracellular coiled-coil domain (CC) and a C-terminal glycosylphosphatidyl inositol (GPI) anchor [20]. This unique structure allows BST2 to insert one end into the viral lipid bilayer and the other end into the cell membrane [28, 30] to prevent virus release. BST2 protein is modified by N-linked glycosylation, and dimerizes through disulfide bonds formed between cysteine residues [20, 28]. The glycosylation and dimerization are also important for the antiviral function of BST2 [28].

BST2 also inhibits the release of other enveloped viruses, including simian immunodeficiency virus (SIV) [13], murine leukemia virus (MLV) [11], Lassa virus and Marburg virus [29], Ebola virus [17, 18], Kaposi’s sarcoma-associated herpesvirus (KSHV) [24], Influenza A Viruses (FLUAV) [10] and hepatitis B virus (HBV) [23]. Viruses have evolved to overcome the restriction of BST2. HIV-1 uses Vpu [26, 32], HIV-2 uses envelope glycoprotein [21], while different SIVs use Nef or (and) envelope glycoproteins [13, 15, 38] to counteract BST2. Ebola glycoprotein [18] and KSHV K5 protein [24, 27] can also antagonize BST2. These aforementioned viral proteins cause BST2 downregulation on the cell surface, thus facilitating viral release. Recently, it has been reported that hemagglutinin (HA) and neuraminidase (NA) are important for pandemic FLUAV to antagonize BST2 [10]. HBV HBx protein inactivates BST2 antiviral function specifically in hepatocytes [23].

Orthologues of BST-2 in non-primate mammals, including mouse [11, 22], sheep [1], pig [25], cat [5, 6, 8], dog [34, 35] and horse [37] also have been reported to possess antiviral activity. Takeda et al. identified three isoforms of bovine BST2 (bBST2): bBST2A1, bBST2A2 and bBST2B. bBST2A1 and bBST2A2 are highly homologous in amino acid sequences, and both harbor an N-linked glycosylation site and a GPI-anchoring motif, while bBST2B has neither of them. Exogenous expression of bBST2A1 or bBST2A2 markedly inhibits bovine leukemia virus (BLV) and vesicular stomatitis virus (VSV), while the antiviral activity of bBST2B was much weaker [31]. However, the influence of bBST2 on the replication of other retroviruses remains unstudied. In this study, we investigate the antiviral activity of human and bovine BST2 against retroviruses that infect human or cattle. Like human BST2 (hBST2), bBST2A1 suppresses virus release. The GPI anchor and dimerization are indispensable for the antiviral activity of bBST2A1. However, while hBST2 inhibits cell-to-cell transmission of prototypic foamy virus (PFV), bovine foamy virus (BFV) and bovine immunodeficiency virus (BIV), bBST2A1 is inactive against cell-to-cell infection of these viruses.

## Methods

### Plasmids

BST2 expressing constructs were generated through inserting the coding sequences of bovine BST2A1 and human BST2 with an N-terminal HA tag into pQCXIP vector (Clontech). HIV-1 molecular clone NL4–3.E-U- was generated by mutating the start codons of *env* and *vpu* genes in original clone NL4–3. PFV full-length infectious clone pcPFV was kindly provided by Maxine L. Linial (Division of Basic Sciences, Fred Hutchinson Cancer Research Center, Seattle, WA). The pcBIV was constructed by replacing the U3-R region in pBIV127 (provided by Dr. Charles Wood from University of Nebraska Lincoln) 5′ LTR with a CMV promoter. Similarly, pcBFV was created by replacing the U3-R region in pBS-BFV-Z1 (a BFV clone isolated in our lab, unpublished data) 5′ LTR with a CMV promoter. bBST2A1 mutants N101A and C62/72/100A were generated by PCR-based site-direction mutagenesis.

### Cell culture and transfection

Indicator cell lines for BFV (BFVL), PFV (PFVL) and BIV (BIVL) were generated in our lab. BFVL (BHK21-derived indicator cells containing a *luciferase* gene under the control of the BFV LTR) [12], PFVL (BHK21-derived indicator cells containing a *luciferase* gene under the control of the PFV LTR) ([34, 35]; BIVL (BHK21-derived indicator cells containing a *luciferase* gene under the control of the BIV LTR) [36]. These indicator cells, HEK293T and TZM-bl cells were maintained in Dulbecco’s modified Eagle’s medium (high glucose) with 10% fetal bovine serum (Hyclone), 100 U/ml penicillin/streptomycin. HEK293T cells were transfected by using the polyethyleneimine (PEI) reagent (Sigma-Aldrich) [7].

### Antibodies

Anti-Tubulin and anti-HA antibodies were purchased from Santa Cruz Biotechnology (Santa Cruz, CA), anti-HIV-1 p24 antibody from Millipore. BIV, PFV and BFV Gag antibodies were generated in our lab.

### Western blotting

HEK293T cells were transfected with various palsmids. Fourty-eight hours post-transfection, the cells were havested and washed twice with 1 × phosphate-buffered saline, then lysed in the buffer with 50 mM Tris (pH 7.4), 150 mM NaCl, 2 mM EDTA, 3% Glycerol, 1% NP-40 for 30 min on ice. After centrifugation at 13,000×g for 10 min at 4 °C, the supernatants were collected. The samples were separated by 12% polyacrylamide gel electrophoresis (SDS-PAGE) and transferred onto the PVDF membrane (Millipore) by electroblotting for 1 h at 100 V, 4 °C. The membranes were blocked in 5% non-fat milk (in 1 × phosphate-buffered saline) for 45 min at room temperature, and probed with the indicated primary antibodies for 90 min at room temperature. After incubation with either goat anti-mouse or goat anti-rabbit secondary antibody, the membranes were treated with the enhanced chemiluminescence reagents (Millipore), the protein signals were detected by exposure to X-ray films.

### Luciferase assays

Transfected or infected cells were collected and lysed in cell culture lysis buffer (Promega). Luciferase activity was measured using the luciferase assay system (Promega).

## Results

### bBST2A1 inhibits retrovirus release

**Fig. 1:**
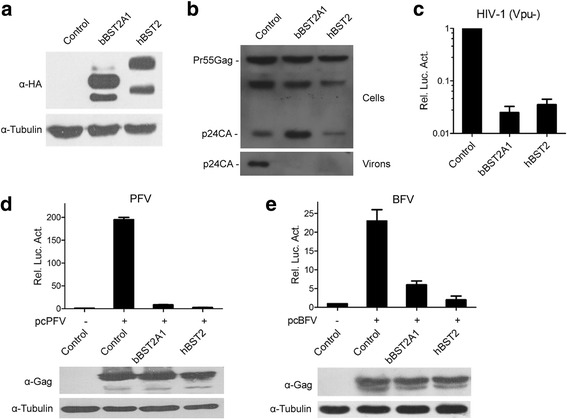
bBST2A1 inhibits release of retroviruses. (**a**) HA-bBST2A1 and HA-hBST2 expressing plasmids were transfected into HEK293T cells. Expression of BST2 orthologues were analyzed by western blotting 48 h post-transfection. (**b**-**c**) HIV-1 molecular clone NL4–3.E-U- and HIV-1 Env expressing plasmids were transfected into HEK293T cells together with vector control, HA-bBST2A1 or HA-hBST2 expressing plasmid. 48 h post-transfection, cells and culture supernatants were subjected to western blotting using HIV-1 p24 antibody (**b**). Supernatants were also used to infect TZM-bl indicator cells, followed by luciferase assays (**c**). (**d**-**e**) HEK293T cells were transfected with pcPFV (**d**) or pcBFV (**e**), together with BST2 plasmids or vector control. 48 h post-transfection, cells were harvested for western blotting, and supernatants were collected and used to infect PFVL (d) or BFVL (**e**) indicator cells. Data of three independent experiments are summarized in the bar graph. Data are represented as mean ± SEM

bBST2A1 and bBST2A2 are highly homologous and possess similar antiviral activity against BLV and VSV [31]. We have cloned a bBST2 gene from cDNA of fetal bovine lung (FBL) cells [34, 35], and this gene turns out to be bBST2A1. bBST2A1 and hBST2 were expressed to similar levels in HEK293T cells, tested by western blotting (Fig. 1a). Then we tested the antiviral activity of bBST2A1 against *vpu*-deficient HIV-1. HEK293T cells were transfected with a *vpu*-deficient HIV-1 molecular clone pNL4–3.E-U-, a plasmid expressing HIV-1 Env, together with a plasmid expressing bBST2A1 or hBST2. Western blotting results showed that both BST2s reduced HIV-1 virions released in culture supernatants (Fig. 1b). We noted that hBST2 also slightly reduced the level of cell-associated p24, whereas the expression of bBST2A1 increased the level of cell-associated p24 (Fig. 1b). Virion release was also evaluated by infecting HIV indicator cells TZM-bl. As expected, the release of *vpu*-deficient HIV-1 was strongly inhibited by BST2s (Fig. 1c).

We also tested the effect of BST2s on foamy viruses, including PFV and BFV. Western blotting results showed that BST2s did not reduce the cell-associated FV Gag levels (Fig. 1d and e). Since the foamy viruses produced in culture supernatants were present at concentrations too low for western blotting, FV release was assessed by infecting indicator cell lines. We observed markedly decrease of cell-free FVs produced upon the expression of BST2s (Fig. 1d and e). These results indicated bBST2A1 inhibits the release of retroviruses that infect human or cattle.

### Dimerization and GPI anchor are important for the antiviral function of bBST2A1

**Fig. 2:**
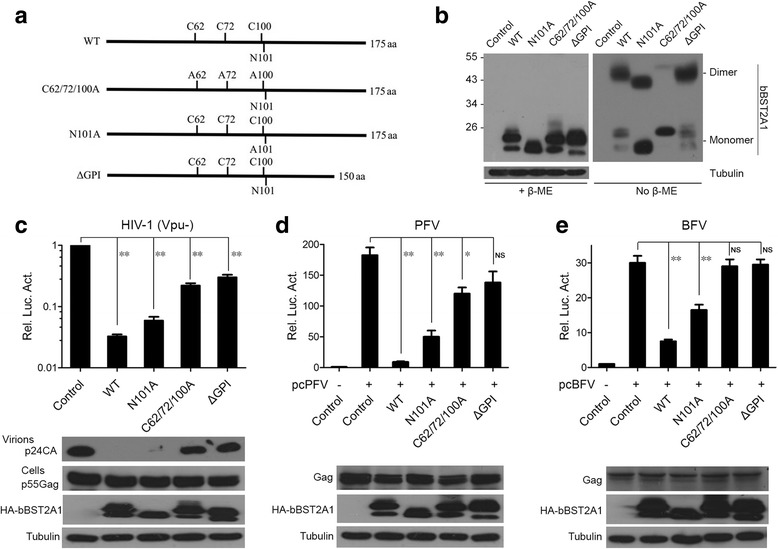
Dimerization and GPI anchor are critical for antiviral activity of bBST2A1. (**a**) Schematic representations of bBST2A1 mutants. (**b**) Western blotting analysis (anti-HA) of HEK293T cells transfected with plasmids expressing wild-type (WT) and mutant bBST2A1. Samples were untreated or treated with β-mercaptoethanol (β-ME) prior to analysis. Numbers to the left of (**b**) represent the positions and sizes (in kDa) of molecular weight markers. (C-E) HEK293T cells were transfected with NL4–3.E-U- (and HIV-1 Env) (**c**), pcPFV (**d**) or pcBFV (**e**), together with wild-type (WT) or mutant bBST2A1 plasmids. 48 h post-transfection, viral supernatants were collected and used to infect TZM-bl (**c**), PFVL (**d**) and BFVL (**e**). Luciferase assays were performed 48 h post-infection. HIV-1 virions containing culture supernatants were also subjected to western blotting using p24 antibody (**c**). Cells were harvested for western blot using indicated antibodies. Data of three independent experiments are summarized in the bar graph. Data are represented as mean ± SEM. Significant differences between the control and bBST2A1s values were determined using the student’s t test. The threshold for significance was set at *p* < 0.05. *p < 0.05, **p < 0.01, NS: not significant, *P* > 0.05

It has been reported that glycosylation, dimerization and GPI anchor are important for the antiviral function of hBST2 [28]. To test if these modifications are also important for bBST2A1, we generated mutants deficient in glycosylation, dimerization and GPI anchor addition. It has been reported that bBST2A1 harbors an N-linked glycosylation site N101 [31]. To defect the glycosylation modification, we substituted N101 with an A (alanine) (Fig. 2a). Cysteines at hBST2 position 53, 63 and 91 form disulfide bonds with an orthologous cysteine, and the triple cysteine mutation C53/63/91A abolished the ability of hBST2 to form disulfide-linked dimers [28]. We also found three cysteines in bBST2A1 at position 62, 72 and 100 in extracellular domain, and generated the triple cysteine mutant C62/72/100A (Fig. 2a). The putative GPI-recognition site in bBST2A1 is N152 [31], and we generated ΔGPI mutant by deleting the C-terminal 25 aa (Fig. 2a). The expression of wild-type (WT) and mutant bBST2A1 were analyzed by western blotting (Fig. 2b). WT bBST2A1 was detected as double bands, indicating the glycosylated and non-glycosylated forms. N101A was non-glycosylated and detected as a single band (Fig. 2b, +β-ME). The triple cysteine mutant (C62/72/100A) abolished the ability of tetherin to form β-mercaptoethanol (β-ME)-sensitive dimers (Fig. 2b). Then we tested the effects of these mutants on retrovirus release. All three mutants led to a decrease in restriction of virus release (Fig. 2c-e). The C62/72/100A and ΔGPI mutants lost most of the antiviral activity, while N101A mutation only slightly impaired the inhibitory activity (Fig. 2c-e). Western blotting results in Fig. 2c also showed C62/72/100A and ΔGPI mutation impaired the function of bBST2A1 to block virus release. These data demonstrate that dimerization and GPI anchor are indispensable for bBST2A1 antiviral activity.

### bBST2A1 does not inhibit cell-to-cell infection of BIV, PFV and BFV

**Fig. 3:**
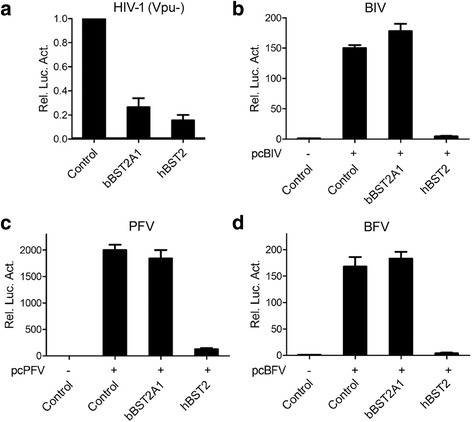
bBST2A1 does not inhibit cell-to-cell transfection of BIV, PFV and BFV. HEK293T cells were transfected with NL4–3.E-U- (and HIV-1 Env) (**c**), pcPFV (**d**) or pcBFV (**e**), together with vector control or BST2 plasmids. 48 h post-transfection, cells were harvested and 10% of the transfected 293 T cells were used to co-culture with TZM-bl (*a*), BIVL (**b**), PFVL (**c**) and BFVL (d)(100,000 indicator cells). 48 h later, luciferase assay was performed. Data of three independent experiments are summarized in the bar graph. Data are represented as mean ± SEM

It has been reported that hBST2 can also inhibit HIV-1 cell-to-cell transmission besides blocking virus release [2, 9, 19]. We also tested whether bBST2A1 inhibited cell-to-cell infection. HEK293T cells were transfected with molecular clones of different viruses, together with BST2-expressing plasmids. The transfected cells were collected and used to co-culture with corresponding indicator cell lines. BST2s also suppressed cell-to-cell infection of *vpu*-deficient HIV-1 (Fig. 3a), although the effect was not as strong as on virus release (Fig. 1c). To our surprise, bBST2A1 has little influence on cell-to-cell spread of BIV, PFV and BFV, while hBST2 still inhibits cell-to-cell infection of these viruses (Fig. 3b-d).

## Discussion

Human BST2 has been identified as a host restriction factor that blocks the release of a range of enveloped viruses, and BST2s from different species all possess antiviral activity [1, 5, 6, 8, 11, 22, 25, 31, 33, 37]. Three isoforms of bovine BST2 have been identified, termed bBST2A1, bBST2A2 and bBST2B. bBST2A1 and bBST2A2 significantly inhibited BLV and VSV [31]. Here, we have tested the antiviral activity of bBST2A1 against retroviruses that infect human or cattle, and found that bBST2A1 is capable to inhibit virus release of HIV-1, PFV and BFV. Our data have broadened the antiviral spectrum of bovine BST2.

bBST2A1 and bBST2A2 are glycosylated and appended with a GPI anchor, while bBST2B has neither of these modifications. The antiviral activity of bBST2B against BLV and VSV is much weaker than that of bBST2A1 and bBST2A2 [31], which suggests that glycosylation and/or GPI anchor are crucial for bovine BST2 to inhibit viruses. In this study, we have generated bBST2A1 mutants that are deficient in glycosylation and GPI anchor addition. ΔGPI mutant lost most of the antiviral activity, while N101A mutant that is glycosylation defective still maintained the inhibitory activity (Fig. 2). hBST2 dimerizes through the disulfide bonds formed between orthologous cysteines at positions 53, 63 and 91 in extracellular domain [28]. We found three cysteines in extracellular domain of bBST2A1 at position 62, 72 and 100. To test whether these cysteines are critical for bBST2A1 dimerization, cysteines (C) at 62, 72 and 100 were replaced with alanines (A). We found that C62/72/100A mutant was deficient in dimerization and lost the antiviral activity (Fig. 2). Our data have demonstrated that, like hBST2, GPI anchor and dimerization are essential for antiviral activity of bBST2A1.

Cell-to-cell transmission is a more efficient mean for viral dissemination than cell-free infection. Actually, FVs and BIV transmit mainly through cell-cell contact. The effect of hBST2 on cell-to-cell transmission is controversial [2–4, 9, 14, 16, 19]. In a study from Dietrich et al., feline BST2 restricts the release of feline immunodeficiency virus (FIV), but does not suppress FIV cell-to-cell spread [6]. Here, we show that hBST2 suppressed cell-to-cell infection of HIV-1 (Vpu-), BIV, PFV and BFV. bBST2A1 also inhibited HIV-1 (Vpu-) cell-to-cell transmission, but exhibited no inhibitory activity on cell-to-cell transmission of BIV, BFV and PFV (Fig. 3). Our data suggest that BST2s from different species may possess similar activity on virus release, but differ in their effect on virus cell-to-cell transmission. bBST2A1 showed different effect on cell-to-cell transmission of different viruses, suggesting viruses may usurp different machineries on cell-to-cell transmission. Further studies are required to clarify the roles of different BST2s on virus cell-to-cell transmission and demonstrate the mechanisms of action.

## Conclusions

In this study, we investigated the antiviral activity of bovine BST2A1 against retroviruses HIV-1, BIV, BFV and PFV. bBST2A1 blocked the release of HIV-1, BFV and PFV, but not the cell-to-cell infection of BIV, BFV and PFV. We also found that GPI anchor and dimerization of bBST2A1 were crucial for blocking virus release.

## Abbreviations

BFV: Bovine foamy virus
BIV: Bovine immunodeficiency virus
BST2: Bone marrow stromal cell antigen 2
HIV-1: Human immunodeficiency virus type 1
PFV: Prototypic foamy virus
